# Surface Filth as a Medium of Disease

**Published:** 1887-01

**Authors:** 


### Surface Filth as a Medium of Disease

Was the subject of a paper by Rev. Henry A. Wales, of Big Rapids. He said: In 1876, Dr. Henry Bowditch, of Boston, made an estimate of the annual cost to the people of the United States because of unnecessary sickness, and placed the figures at $100,000,000. Later, one of our own physicians—who is with us to-day—revised the estimate, going more into detail, and he increased the amount to $300,000,000; a loss each year of over $10,000,000 to the people of Michigan. And this estimate leaves out of view the physical suffering, the mental pain and anguish, and the death of loved ones around our social circles. In regard to filth he said: By surface filth we mean anything of this disgusting nature that is thrown upon or is suffered to lie upon the surface of the ground around our habitations or places of business—garbage, or the refuse of vegetable and animal matter; dirty water of every description, from that in which food is cleansed, to the dish-water and slops of a household; dirt swept from the floors of the home or shop; the contents of the wood-box and spittoons; the refuse of wood-yard, hen-house or pig-pen; and the excrement from chambers and privies. Besides these, there are sources of filth in musty rooms, woolen blankets, feather-beds, and the cellars under the house. Rubbish of any kind always becomes filthy if allowed to stand; and dampness increases filth by causing fermentation and vegetable growth. A close inspection of all the premises of a habitation is continuously needed, that nothing which may cause filth shall be allowed to accumulate. Mr. Wales thought that the "grand march of the giant contagion begins in the surface filth and the vaults of the civilized privy;" and he thought that if the dry earth system was universally introduced, it would exterminate such diseases as cholera, dysentery, and typhoid fever, as they are propagated solely by germs in the voided excrement.

